# The prognostic value of PD‐L1 expression in upper tract urothelial carcinoma varies according to platelet count

**DOI:** 10.1002/cam4.1686

**Published:** 2018-07-31

**Authors:** Yu Miyama, Teppei Morikawa, Jimpei Miyakawa, Yuichi Koyama, Taketo Kawai, Haruki Kume, Masashi Fukayama

**Affiliations:** ^1^ Department of Pathology Graduate School of Medicine The University of Tokyo Tokyo Japan; ^2^ Department of Diagnostic Pathology NTT Medical Center Tokyo Tokyo Japan; ^3^ Department of Urology Graduate School of Medicine The University of Tokyo Tokyo Japan

**Keywords:** host–tumor interaction, immune checkpoint inhibitor, serum marker, upper urinary tract carcinoma, urothelial cancer

## Abstract

Programmed cell death ligand‐1 (PD‐L1) is a ligand for programmed cell death‐1 (PD‐1) that negatively regulates T‐cell activation and plays a crucial role in suppressing anti‐tumor host immunity. Although PD‐L1 is a promising immunotherapy target in various cancers, including urothelial carcinoma (UC), the prognostic significance of PD‐L1 in UC is unclear. As platelets help protect tumor cells from immune elimination in the circulatory system, we hypothesized that tumor PD‐L1 and circulating platelets might synergistically promote tumor metastasis, and that the prognostic significance of PD‐L1 might vary according to platelet count. We immunohistochemically examined tumor PD‐L1 expression in 271 patients with upper tract UC, which revealed PD‐L1 positivity in 31 of 271 cases (11%). The associations of tumor PD‐L1 expression with outcomes varied among patients with high or low platelet counts (*P*_interaction_ < 0.004). Among patients with high platelet counts (N = 136), PD‐L1 positivity (N = 15) was significantly associated with shorter metastasis‐free survival (univariate hazard ratio [HR]: 6.23, 95% confidence interval [CI]: 2.95‐13.1; multivariate HR: 2.68, 95% CI: 1.27‐5.64) and shorter overall survival (univariate HR: 4.92, 95% CI: 2.14‐11.3, multivariate HR: 2.78, 95% CI: 1.19‐6.51). In contrast, among patients with low platelet counts (N = 135), PD‐L1 positivity (N = 16) was not significantly associated with these outcomes. Our results suggest that tumor PD‐L1 expression and platelet count might interact and help regulate tumor progression. Although a larger prospective study is needed to validate our findings, this relationship is important to consider, as immunotherapies targeting the PD‐1/PD‐L1 axis have gained significant attention as promising therapies for UC.

## 1 INTRODUCTION

Programmed cell death ligand‐1 (PD‐L1) is a ligand for programmed cell death‐1 (PD‐1) that negatively regulates T‐cell activation and plays a crucial role in suppressing anti‐tumor host immunity.^1^ Antibodies that interfere with the interaction between PD‐L1 and PD‐1 have significant clinical activity in various malignancies, including advanced urothelial carcinoma (UC).^2^, ^3^, ^4^, ^5^ Cases of UC can be categorized according to whether the UC involves the lower tract (bladder and urethra) or upper tract (UTUC: ureter and renal pelvis), with the latter category accounting for 5%‐10% of all UC cases.^6^ Atezolizumab is a blocking antibody that targets PD‐L1, and provided a higher objective response rate in cases of UTUC (39%) than in cases of lower tract UC (17%) during a clinical trial of first‐line treatment for cisplatin‐ineligible patients with locally advanced or metastatic UC.^7^ That result suggests that targeting the PD‐1/PD‐L1 axis may be more effective in UTUC. However, several other studies have revealed inconsistent results regarding the association between tumor PD‐L1 positivity and prognosis in cases of UC.^8^, ^9^, ^10^, ^11^, ^12^, ^13^, ^14^, ^15^, ^16^, ^17^ Therefore, it is important to improve our understanding of the prognostic significance of PD‐L1 expression in UC, in order to better select patients who are likely to respond to PD‐1/PD‐L1 blockade.

Accumulating evidence suggests that the prognostic value of tumor biomarkers may vary according to the patient's characteristics, such as physical activity and aspirin use, through host–tumor interactions.^18^, ^19^, ^20^, ^21^ For example, a recent study revealed that postdiagnosis use of aspirin was associated with better survival among patients with PD‐L1‐low colorectal cancer, but not among patients with PD‐L1‐high tumors.^21^ Furthermore, aspirin's effects on the metastatic process may depend on its inhibition of platelet function.^22^ In this context, platelets can protect tumor cells from immune elimination in the circulatory system, promote tumor cell arrest within the vasculature, and affect tumor cell survival, which can support the establishment of secondary lesions.^23^ As both PD‐L1 and platelets promote tumor cell survival through immune suppression, we hypothesized that tumor PD‐L1 expression and circulating platelet might synergistically promote tumor metastasis, and that the prognostic significance of PD‐L1 positivity might vary according to platelet count. Therefore, we examined the interactive effect of tumor PD‐L1 and platelet count on the prognosis of 271 patients with UTUC.

## 2 PATIENTS AND METHODS

### 2.1 Study population

This retrospective study evaluated data from 271 patients with UTUC who underwent nephroureterectomy at The University of Tokyo Hospital between 1990 and 2017. All research protocols for this study were approved by the institutional review board of The University of Tokyo (3124). Cases were excluded if they involved neoadjuvant chemotherapy or distant metastasis at the time of diagnosis, and none of the patients had received immune checkpoint blockade therapy. Preoperative platelet counts had been assessed within 30 days before the nephroureterectomy, and the patients were divided into platelet‐high and platelet‐low groups based on the median value (234 × 10^9^/L).

### 2.2 Histopathological evaluation

Hematoxylin and eosin‐stained slides from all cases were reviewed by a single pathologist (TM) who was blinded to the patients’ clinical outcomes. All tumors were histologically diagnosed as UCs. Tumor grade and stage were defined according to the 2016 World Health Organization grading system and the TNM classification system.^24^

### 2.3 Immunohistochemical analysis

Tissue microarrays (TMAs) were constructed as previously described.^25^, ^26^ Core samples were obtained using a 2‐mm‐diameter needle at the center and periphery of the paraffin‐embedded tumor specimens, and the cores were transferred to a recipient paraffin block using a tissue microarrayer (Beecher Instruments Inc., Sun Prairie, WI). As internal positive controls, placental and lymph node tissues were included in each TMA (Figure S1). Preparation of sections from the TMAs was performed as previously described.^27^ Immunohistochemical analysis of PD‐L1 expression was performed using a validated rabbit monoclonal antibody against human PD‐L1 (clone SP263; prediluted; Ventana Medical Systems, Tucson, AZ)^28^, ^29^, ^30^, ^31^ and standard techniques for a Ventana Benchmark XT Autostainer (Ventana Medical Systems). Antigen retrieval was performed using Cell Conditioning Solution (CC1‐buffer; Ventana Medical Systems), and visualization was achieved using the OptiView DAB Universal Kit (Ventana Medical Systems) and hematoxylin counterstaining.

Immunoreactivity was independently assessed by two pathologists (YM and TM) who were blinded to the patients’ clinical outcomes. In cases with discrepant results, the slides were reviewed under a multi‐head microscope and discussed to determine the final score. The estimated percentage of tumor cells exhibiting partial or complete membranous staining was recorded. PD‐L1 expression was evaluated at both the tumor center and tumor periphery, and the higher percentage of PD‐L1 expression was selected as the final score for analysis. Cases were classified as positive for PD‐L1 expression using a cutoff value of 5%.^4^, ^9^, ^10^, ^11^, ^12^, ^13^, ^16^

### 2.4 Statistical analysis

All statistical analyses were performed using SAS software (version 9.3; SAS Institute, Cary, NC), and all *P*‐values were two‐sided. Multiple hypothesis testing was performed using *P*‐values that were adjusted via Bonferroni's correction to *P *=* *0.0045 (0.05/11). Categorical data were analyzed using the chi‐square test or Fisher's exact test (for tumor grade and lymph node metastasis). Metastasis‐free survival (MFS), and overall survival (OS) was analyzed using the Kaplan–Meier method and log‐rank test. Univariate and multivariate Cox proportional hazard regression models were used to control for confounding variables. The multivariate Cox regression models initially included sex, age at diagnosis, tumor side, tumor location, history of bladder cancer, tumor grade, concomitant carcinoma in situ, lymphovascular invasion, tumor stage, lymph node metastasis, and platelet count. Backward elimination was performed using a threshold of *P *=* *0.05 to select variables for the final model. An interaction was assessed using the Wald test for the cross‐product of PD‐L1 positivity and platelet count in a multivariate Cox model.

## 3 RESULTS

### 3.1 Clinicopathological significance of PD‐L1 positivity in UTUC

**Figure 1:**
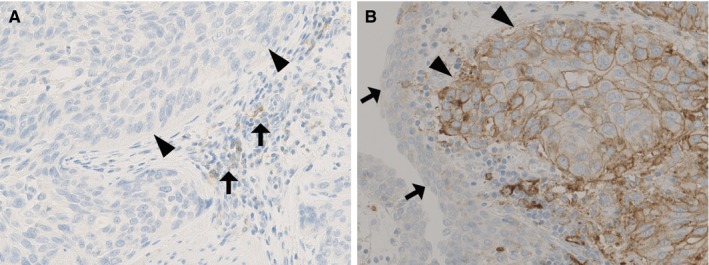
PD‐L1 expression in upper tract urothelial carcinoma. A, Negative PD‐L1 expression on tumor cells (arrowheads). Some immune cells are stained for PD‐L1 (arrows). B, Positive PD‐L1 expression on tumor cells (arrowheads), with a surface that is covered by PD‐L1‐negative non‐neoplastic urothelium (arrows)

Representative photomicrographs of the PD‐L1 immunohistochemistry results are shown in Figures 1 and S1. We detected PD‐L1 positivity in 31 of 271 cases (11%), although the benign urothelium in the TMAs was never positive for PD‐L1. There was a strong correlation between PD‐L1 positivity at the tumor's center and periphery (correlation coefficient = 0.76, *P *<* *0.0001). The associations between PD‐L1 positivity and the patients’ clinicopathological features are summarized in Table 1. PD‐L1 positivity was significantly associated with lymphovascular invasion (*P *=* *0.001) and a higher tumor stage (*P *<* *0.0001), but was not associated with platelet count.

**Table 1:** Associations of PD‐L1 positivity with clinicopathological features of patients with upper tract urothelial carcinoma who underwent nephroureterectomy

		PD‐L1 expression		
	Total N	Negative	Positive	*P*
All cases	271	240 (89%)	31 (11%)	
Sex				
Male	192	169 (88%)	23 (12%)	0.66
Female	79	71 (90%)	8 (10%)	
Age, y				
<70	136	123 (90%)	13 (10%)	0.33
≥70	135	117 (87%)	18 (13%)	
Side				
Left	135	118 (87%)	17 (13%)	0.55
Right	136	122 (90%)	14 (10%)	
History of bladder cancer				
No	224	196 (88%)	28 (13%)	0.23
Yes	47	44 (94%)	3 (6%)	
Tumor location				
Renal pelvis	162	140 (86%)	22 (14%)	0.18
Ureter	109	100 (92%)	9 (8%)	
Tumor grade				
Low	41	40 (98%)	1 (2%)	0.060
High	230	200 (87%)	30 (13%)	
Lymphovascular invasion				
Absent	169	158 (93%)	11 (7%)	0.0010
Present	102	82 (80%)	20 (20%)	
Concomitant carcinoma in situ				
Absent	142	129 (91%)	13 (9%)	0.22
Present	129	111 (86%)	18 (14%)	
Tumor stage				
pTa/pTis	84	84 (100%)	0 (0%)	
pT1	53	49 (92%)	4 (8%)	
pT2	24	23 (96%)	1 (4%)	<0.0001
pT3	104	83 (80%)	21 (20%)	
pT4	6	1 (17%)	5 (83%)	
Lymph node metastasis				
Absent	248	223 (90%)	25 (10%)	0.034
Present	23	17 (74%)	6 (26%)	
Median platelet count ± SD, ×10^9^/L	234 ± 77	236 ± 77	228 ± 75	0.97
Platelet count, ×10^9^/L				
<234	135	119 (88%)	16 (12%)	0.83
≥234	136	121 (89%)	15 (11%)	

### 3.2 PD‐L1 positivity and clinical outcomes in cases of UTUC

**Figure 2:**
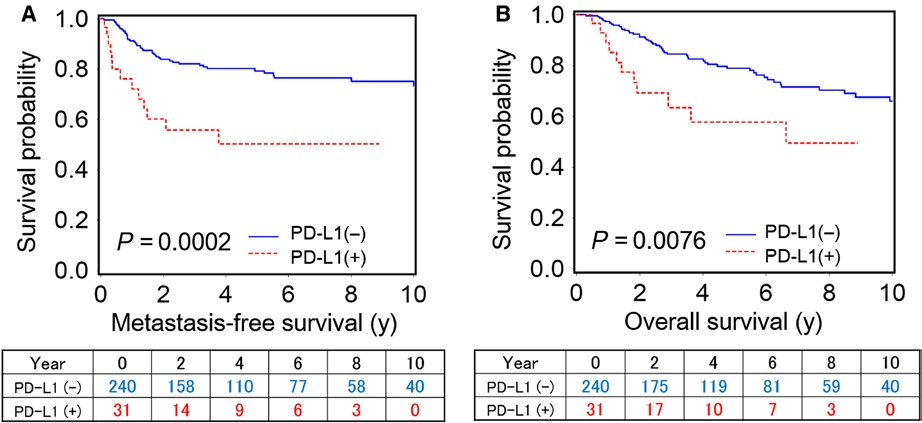
Kaplan–Meier curves for metastasis‐free survival (A) and overall survival (B) after nephroureterectomy according to PD‐L1 positivity in upper tract urothelial carcinoma

Among the 271 patients with UTUC who underwent nephroureterectomy, 59 patients developed metastasis and 65 patients died during a median follow‐up of 52 months (interquartile range: 24‐100 months with censoring). The Kaplan–Meier curves for the patients’ clinical outcomes according to PD‐L1 positivity are shown in Figure 2. In the univariate analyses, PD‐L1 positivity was significantly associated with shorter MFS (log‐rank *P *=* *0.0002) and shorter OS (log‐rank *P *=* *0.0076). However, in the multivariate Cox proportional hazards regression analyses, PD‐L1 positivity was not an independent predictor of poor outcomes (Table 2). Tumor stage and lymph node status were major confounders (Tables S1 and S2). Platelet count was not significantly associated with MFS or OS (Tables S1 and S2; Figure S2).

**Table 2:** PD‐L1 positivity and outcomes among patients with upper tract urothelial carcinoma

		Metastasis‐free survival			Overall survival		
	Number of cases	Number of events	Univariate HR (95% CI)	Multivariate HR (95% CI)^a^	Number of events	Univariate HR (95% CI)	Multivariate HR (95% CI)^a^
All cases							
PD‐L1 (−)	240	46	1 (reference)	1 (reference)	54	1 (reference)	1 (reference)
PD‐L1 (+)	31	13	3.07 (1.65‐5.70)	1.72 (0.92‐3.24)	11	2.38 (1.23‐4.58)	1.29 (0.66‐2.52)
*P*‐value			0.0004	0.092		0.0097	0.45
Platelet‐low							
PD‐L1 (−)	119	23	1 (reference)	1 (reference)	32	1 (reference)	1 (reference)
PD‐L1 (+)	16	4	1.37 (0.48‐3.89)	0.89 (0.31‐2.59)	4	1.16 (0.41‐3.25)	0.60 (0.21‐1.71)
*P*‐value			0.55	0.83		0.79	0.34
Platelet‐high							
PD‐L1 (−)	121	23	1 (reference)	1 (reference)	22	1 (reference)	1 (reference)
PD‐L1 (+)	15	9	6.23 (2.95‐13.1)	2.68 (1.27‐5.64)	7	4.92 (2.14‐11.3)	2.78 (1.19‐6.51)
*P*‐value			<0.0001	0.0097		0.0002	0.019
*P* for interaction^b^			0.0037	0.0029		0.0025	0.0013

CI, confidence interval; HR, hazard ratio.

a The multivariate Cox regression models initially included sex, age at diagnosis, tumor side, tumor location, history of bladder cancer, tumor grade, concomitant carcinoma in situ, lymphovascular invasion, tumor stage, lymph node metastasis, and platelet count (dichotomized using the median value). Backward elimination was performed using a threshold of *P *=* *0.05 to select variables for the final model.

b The interaction was assessed using the Wald test and the cross‐product of PD‐L1 and platelet count (as a continuous variable) in the Cox model.

### 3.3 Prognostic significance of PD‐L1 positivity according to platelet count

**Figure 3:**
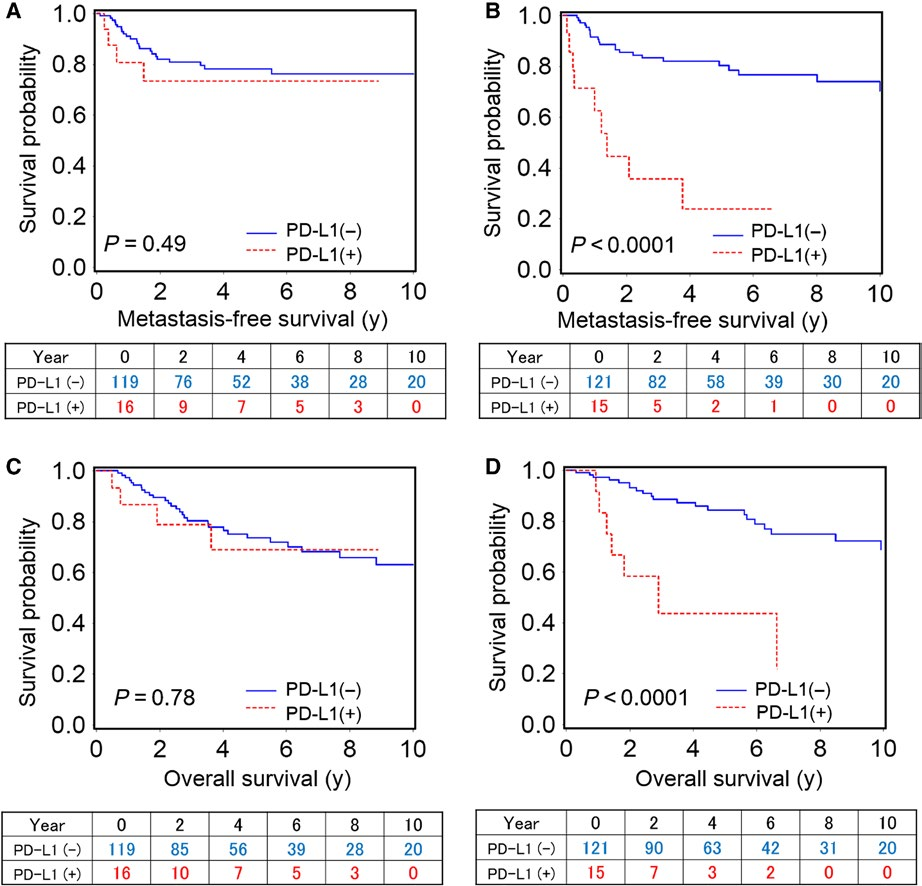
Kaplan–Meier curves for metastasis‐free survival according to PD‐L1 positivity among patients with low platelet counts (A) and high platelet counts (B). Overall survival according to PD‐L1 positivity among patients with low platelet counts (C) and high platelet counts (D)

We detected a significant modifying effect for preoperative platelet count on the relation between PD‐L1 positivity and patient outcomes (*P*_interaction_ < 0.004). Among patients with high platelet counts (N = 136), PD‐L1 positivity (N = 15) was significantly associated with shorter metastasis‐free survival (univariate hazard ratio [HR]: 6.23, 95% confidence interval [CI]: 2.95‐13.1; multivariate HR: 2.68, 95% CI: 1.27‐5.64) and shorter overall survival (univariate HR: 4.92, 95% CI: 2.14‐11.3, multivariate HR: 2.78, 95% CI: 1.19‐6.51). In contrast, among patients with low platelet counts (N = 135), PD‐L1 positivity (N = 16) was not significantly associated with these outcomes (Table 2). The differential effect of PD‐L1 positivity on patient outcomes according to platelet count was also observed in the Kaplan–Meier analyses (Figure 3).

## 4 DISCUSSION

The results from the present study suggest that platelet count can affect the prognostic significance of PD‐L1 positivity in UTUC. In particular, PD‐L1 positivity was significantly associated with shorter MFS and OS among patients with high platelet counts, although PD‐L1 positivity was not significantly associated with prognosis among patients with low platelet counts. To the best of our knowledge, this is the first study to assess the prognostic values of both tumor PD‐L1 expression and platelet count among cancer patients. Our results support an interactive effect of tumor PD‐L1 and platelet count in the regulation of UTUC progression.

There are conflicting data regarding the prognostic significance of PD‐L1 expression in UC. Some studies have revealed that PD‐L1 positivity was associated with a poor prognosis among patients with urinary bladder UC,^8^, ^9^, ^10^, ^15^ while other groups failed to detect a relationship between PD‐L1 positivity and prognosis.^11^, ^12^, ^14^ In addition, three studies have examined the prognostic significance of PD‐L1 positivity in UTUC. Skala et al^13^ did not detect a correlation between PD‐L1 positivity and cancer‐specific survival, while Zhang et al^16^ reported that PD‐L1 positivity on tumor cells was associated with poor cancer‐specific survival. In contrast, Krabbe et al^17^ reported that PD‐L1 positivity was associated with better outcomes among patients with high‐grade organ‐confined UTUC. There are several possible explanations for these discrepancies, such as differences in the cohorts and immunohistochemical methods (eg, antibody clones, detection systems, and positivity cutoffs). Our data suggest that the differential effects of PD‐L1 according to platelet count may also explain the previous discrepant findings.

A host–tumor interaction between platelet count and tumor PD‐L1 status is an intriguing mechanism for the modification of tumor cell behavior. Although no study has examined the potential modifying effect of platelet count in this setting, a recent study revealed that postdiagnosis aspirin use was associated with better survival among patients with PD‐L1‐negative/low colorectal cancer, but not among patients with PD‐L1‐high tumors.^21^ In that context, the effects of aspirin on the metastatic process may depend on the inhibition of platelet function,^22^ which would also support the effect of platelet count on the prognostic value of PD‐L1 expression. Furthermore, accumulating evidence suggests that platelets play crucial roles in tumor metastasis and suppressing anti‐tumor immunity, as they facilitate the generation of circulating tumor cells, protect these cells from immune surveillance, and enhance the intravasation/extravasation of circulating tumor cells.^23^, ^32^, ^33^ Therefore, the immunosuppressive effects of tumor PD‐L1 expression and high platelet counts might synergistically promote tumor metastasis. Nevertheless, further studies are needed to validate our findings and elucidate the mechanisms that explain why tumor PD‐L1 expression differentially affects the biological phenotype of UC cells according to platelet count.

Platelets are potential mediators of anti‐PD‐L1 blockade, as Wang et al^34^ recently reported that conjugating anti‐PD‐L1 antibodies to the surface of platelets could reduce postoperative recurrence and metastasis in mouse models. In those models, removal of the primary tumor promoted migration of the antibody‐conjugated platelets to the surgical site, where they subsequently released their antibodies. This process enhanced the mice's immune response and helped prevent recurrence. Wang et al also demonstrated that the antibody‐conjugated platelets recognized circulating cancer cells before they could develop into metastatic lesions. Based on these experimental data, as well as our finding that patients with tumor PD‐L1 positivity and high platelet counts experienced poor outcomes, it may be possible to utilize platelets during anti‐PD‐L1 therapy (eg, with conjugated antibodies to PD‐L1) in order to prevent postoperative recurrence.

The present study has several limitations. First, the immunohistochemistry was performed using TMA slides, rather than whole‐tissue sections, which may have resulted in underestimated heterogeneity of the tumors’ PD‐L1 positivity. However, TMAs are useful for examining large numbers of samples in standardized conditions and are widely used in studies that have examined PD‐L1 positivity in various cancers.^10^, ^17^, ^35^, ^36^, ^37^, ^38^, ^39^, ^40^ In addition, we systematically constructed the TMAs using large 2‐mm cores from the tumor's center and periphery and detected a significant correlation between PD‐L1 positivity at the two regions in the same tumor. A second limitation is that statistical power was limited due to the relatively small number of patients and events for patients with PD‐L1 positivity. In relation to this limitation, the platelet cutoff value was arbitrarily set to the median value (234 × 10^9^/L) in order to maximize the statistical power of the analyses. In this context, a recent study of UTUC used a similar cutoff value (230 × 10^9^/L),^41^ although other studies have indicated that thrombocytosis (>400 × 10^9^/L) was associated with adverse clinicopathological features and a poor prognosis in cases of UTUC^42^ or urinary bladder UC.^43^ However, this cutoff value was not realistic in the present study, as only a few patients had both PD‐L1 positivity and a platelet count of >400 × 10^9^/L. Thus, future studies are needed to determine the optimal cutoff value for platelet count. A third limitation is the study's retrospective design and the absence of patients who received immune checkpoint blockade therapy, which preclude any conclusions regarding whether PD‐L1 and platelet count are predictive biomarkers in UTUC. A larger prospective study is needed to address these limitations.

In conclusion, PD‐L1 positivity was significantly associated with shorter MFS and shorter OS among patients with UTUC and high platelet counts, although these relationships were not observed among patients with UTUC and low platelet counts. These results suggest that patients’ platelet counts can modify the effect of tumor PD‐L1 status on the behavior of UTUC cells. These results may be clinically useful, given the increasing interest in using immunotherapies that target the PD‐1/PD‐L1 axis to treat patients with UC.

## CONFLICT OF INTEREST

The authors declare no potential conflicts of interest.
