# Supplementary figures and images for: The prognostic value of PD‐L1 expression in upper tract urothelial carcinoma varies according to platelet count
Source: Cancer Med. 2018 Jul 31;7(9):4330–8. doi: 10.1002/cam4.1686 (PMC6143937; doi:10.1002/cam4.1686)

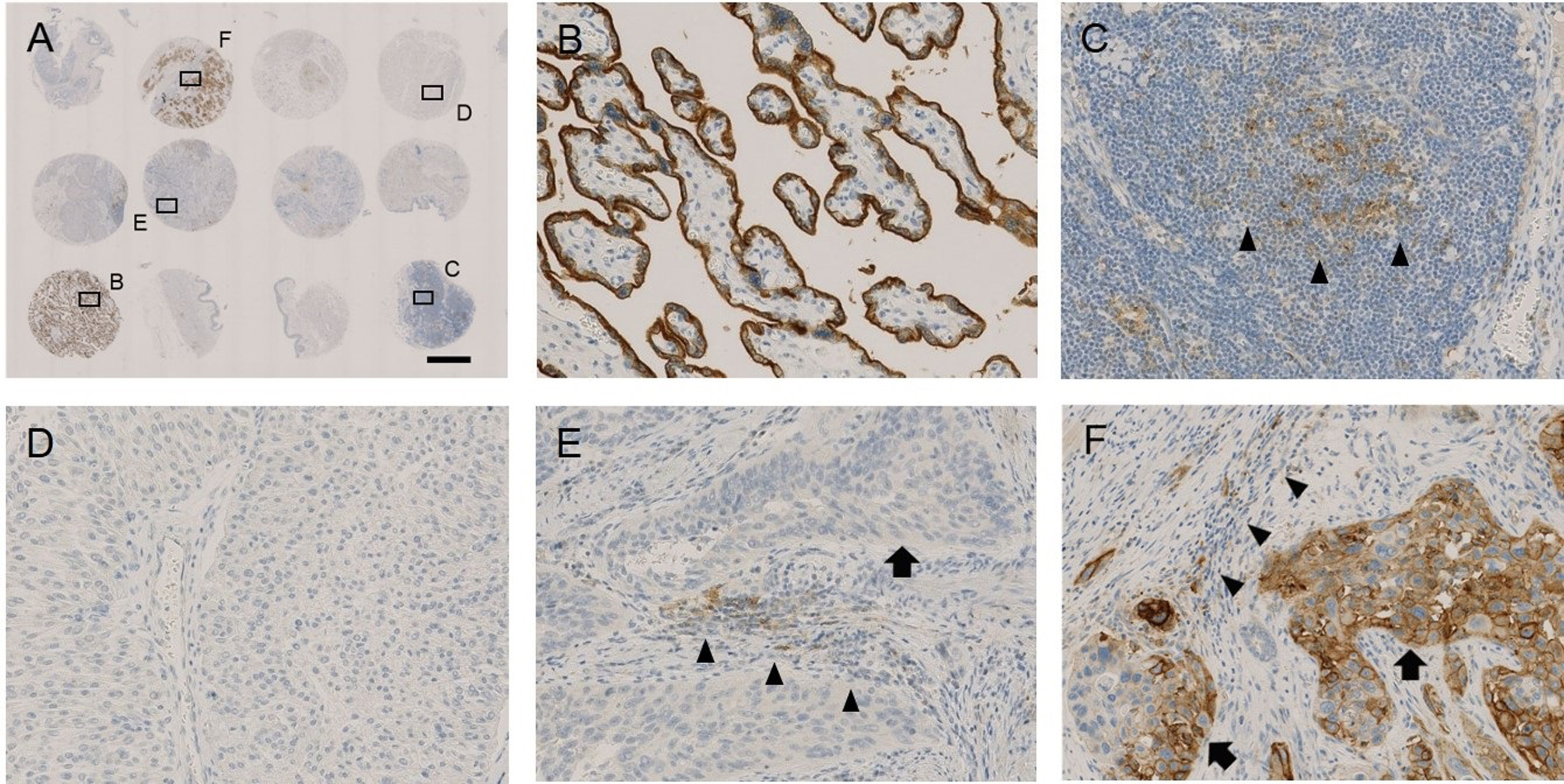

Supplement: Supplementary file 1 [file CAM4-7-4330-s001.tif]

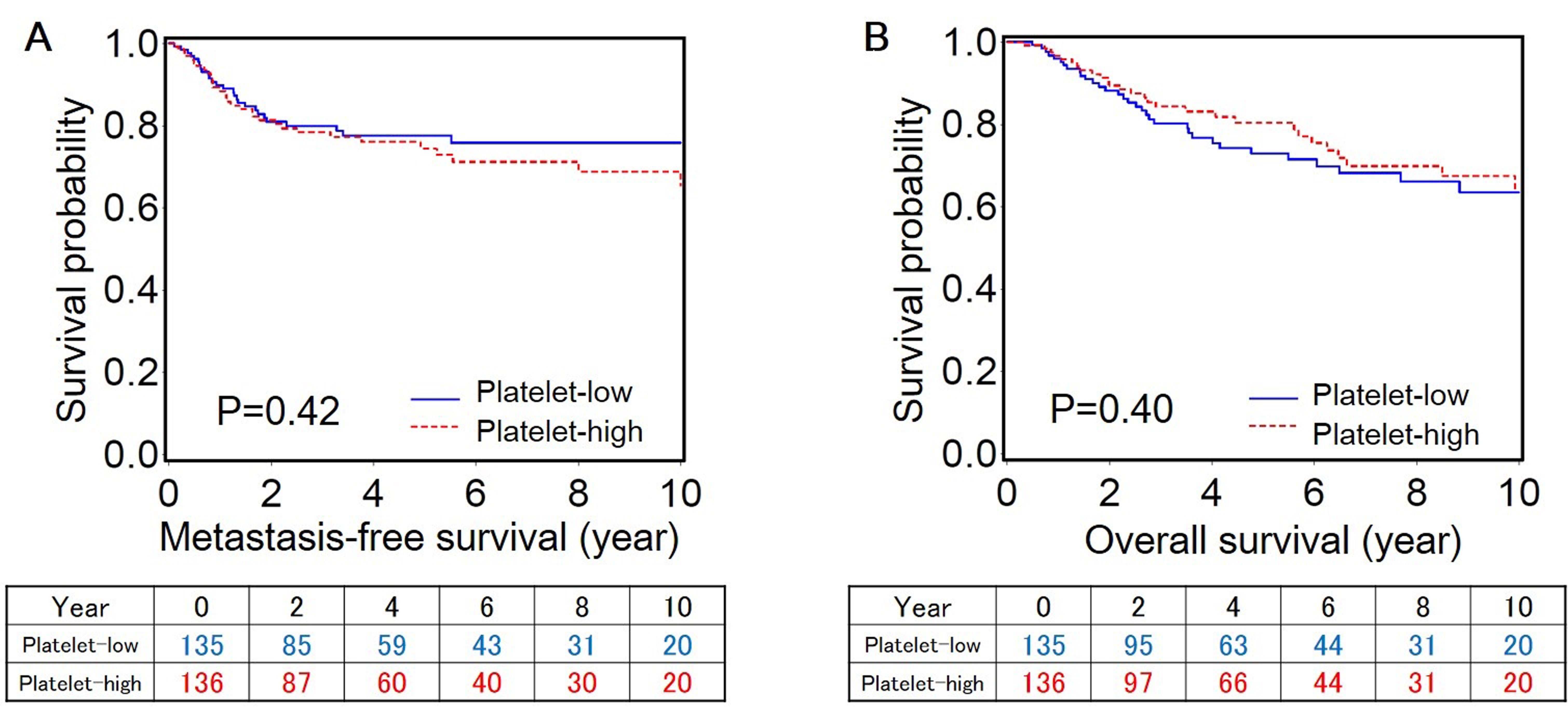

Supplement: Supplementary file 2 [file CAM4-7-4330-s002.tif]
