# Supplementary material for: The prognostic value of PD‐L1 expression in upper tract urothelial carcinoma varies according to platelet count
Source: Cancer Med. 2018 Jul 31;7(9):4330–8. doi: 10.1002/cam4.1686 (PMC6143937; doi:10.1002/cam4.1686)
Supplement: Supplementary file 3 [file CAM4-7-4330-s003.docx]

**Supplementary table 1.** PD-L1 positivity and metastasis-free survival among patients with upper tract urothelial carcinoma

|  | Univariate analysis | |  | Multivariate analysis ^a^ | |
| --- | --- | --- | --- | --- | --- |
|  | HR (95% CI) | *P* |  | HR (95% CI) | *P* |
| PD-L1 expression (positive vs. negative) | 3.07 (1.65–5.70) | 0.0004 |  | 1.72 (0.92–3.24) | 0.092 |
| Platelet count (≥234 vs. <234×10^9^/L) | 1.23 (0.74–2.06) | 0.42 |  |  |  |
| Sex (female vs. male) | 1.19 (0.69–2.04) | 0.53 |  |  |  |
| Age (≥70 vs. <70 years) | 1.83 (1.08–3.08) | 0.024 |  |  |  |
| Side (right vs. left) | 0.70 (0.42–1.18) | 0.18 |  |  |  |
| History of bladder cancer (present vs. absent) | 1.63 (0.91–2.93) | 0.10 |  | 2.42 (1.33–4.40) | 0.0039 |
| Tumor location (ureter vs. renal pelvis) | 1.71 (1.03–2.85) | 0.040 |  |  |  |
| Lymphovascular invasion (present vs. absent) | 5.56 (3.16–9.79) | <0.0001 |  |  |  |
| Concomitant carcinoma *in situ* (present vs. absent) | 2.36 (1.38–4.02) | 0.0017 |  |  |  |
| Tumor stage (≥pT3 vs. ≤pT2) | 11.4 (5.62–23.3) | <0.0001 |  | 9.80 (4.64–20.7) | <0.0001 |
| Lymph node metastasis (present vs. absent) | 5.04 (2.84–8.96) | <0.0001 |  | 2.19 (1.21–3.96) | 0.0096 |

^a^ The multivariate Cox regression models initially included all of the variables in this table. Backward elimination was performed using a threshold of *P* = 0.05 to select variables for the final model. Because patients with low-grade tumors did not develop metastasis, histological grade was not included in the final model.

CI, confidence interval; HR, hazard ratio.

**Supplementary table 2.** PD-L1 positivity and overall survival among patients with upper tract urothelial carcinoma

|  | Univariate analysis | |  | Multivariate analysis ^a^ | |
| --- | --- | --- | --- | --- | --- |
|  | HR (95% CI) | *P* |  | HR (95% CI) | *P* |
| PD-L1 expression (positive vs negative) | 2.38 (1.23–4.58) | 0.0097 |  | 1.29 (0.66–2.52) | 0.45 |
| Platelet count (≥234 vs. <234×10^9^/L) | 0.81 (0.50–1.32) | 0.40 |  |  |  |
| Sex (female vs male) | 0.71 (0.40–1.25) | 0.23 |  |  |  |
| Age (≥70 vs <70y) | 2.54 (1.52–4.24) | 0.0004 |  | 2.30 (1.37–3.84) | 0.0015 |
| Side (right vs left) | 0.76 (0.46–1.24) | 0.27 |  |  |  |
| History of bladder cancer (present vs absent) | 1.00 (0.53–1.92) | 0.99 |  |  |  |
| Tumor grade (high vs low) | 12.4 (1.71–89.1) | 0.013 |  |  |  |
| Tumor location (ureter vs renal pelvis) | 1.26 (0.77–2.06) | 0.35 |  |  |  |
| Lymphovascular invasion (present vs absent) | 4.73 (2.81–7.97) | <0.0001 |  |  |  |
| Concomitant carcinoma *in situ* (present vs absent) | 2.31 (1.39–3.83) | 0.0012 |  |  |  |
| Tumor stage (≥pT3 vs pT2≥) | 6.29 (3.57–11.1) | <0.0001 |  | 4.69 (2.56–8.61) | <0.0001 |
| Lymph node metastasis (present vs absent) | 4.60 (2.63–8.04) | <0.0001 |  | 2.29 (1.27–4.13) | 0.0056 |

^a^ The multivariate Cox regression models initially included all of the variables in this table. Backward elimination was performed using a threshold of *P* = 0.05 to select variables for the final model.

CI, confidence interval; HR, hazard ratio.

**Figure Legend**

**Supplementary Figure 1**. A, PD-L1 immunohistochemistry in upper tract urothelial carcinoma on a tissue microarray, with high-power photomicrographs of the rectangular areas shown in B–F. Bar: 1 mm. B, Placental trophoblasts are diffusely positive for PD-L1. C, PD-L1 expression on dendritic cells from a normal lymph node (arrow heads). D, Negative PD-L1 expression on tumor cells and immune cells. E, Negative PD-L1 expression on tumor cells (arrow), although some immune cells are stained for PD-L1 (arrow heads). F, Positive PD-L1 expression on tumor cells (arrows), with some immune cells that are also stained for PD-L1 (arrow heads).

**Supplementary Figure 2**. Kaplan-Meier curves for metastasis-free survival (A) and overall survival (B) after nephroureterectomy according to platelet count in upper tract urothelial carcinoma.
